# Unmasking latent extrapulmonary tuberculosis with newly diagnosed HIV-1 infection in a COVID-19 patient with prolonged fever

**DOI:** 10.1093/omcr/omac079

**Published:** 2022-07-26

**Authors:** Kaiho Hirata, Koji Watanabe, Takeshi Sasaki, Takashi Yoshimasu, Akihiko Shimomura, Naokatsu Ando, Yasuaki Yanagawa, Daisuke Mizushima, Katsuji Teruya, Yoshimi Kikuchi, Shinichi Oka, Kunihisa Tsukada

**Affiliations:** AIDS Clinical Center, National Center for Global Health and Medicine, Tokyo, Japan; AIDS Clinical Center, National Center for Global Health and Medicine, Tokyo, Japan; 374th Medical Group, Yokota Air Force Base, Tokyo, Japan; Department of Obstetrics and Gynecology, Teine Keijinkai Hospital, Hokkaido, Japan; Department of Breast and Medical Oncology, National Center for Global Health and Medicine, Tokyo, Japan; AIDS Clinical Center, National Center for Global Health and Medicine, Tokyo, Japan; AIDS Clinical Center, National Center for Global Health and Medicine, Tokyo, Japan; AIDS Clinical Center, National Center for Global Health and Medicine, Tokyo, Japan; AIDS Clinical Center, National Center for Global Health and Medicine, Tokyo, Japan; AIDS Clinical Center, National Center for Global Health and Medicine, Tokyo, Japan; AIDS Clinical Center, National Center for Global Health and Medicine, Tokyo, Japan; AIDS Clinical Center, National Center for Global Health and Medicine, Tokyo, Japan

## Abstract

Prolonged fever is a common symptom of COVID-19 infection. However, other febrile diseases continue during the pandemic. Herein, we report a COVID-19-infected patient with prolonged fever despite the lack of oxygen requirement, who was finally diagnosed with tuberculotic lymphadenitis and HIV-1 infection. All symptoms improved rapidly after the initiation of antituberculosis medications. Tuberculosis is an important differential diagnosis for patients with prolonged fever during the COVID-19 pandemic. It is possible that COVID-19 infection could serve to unmask latent infections via a cytokine storm.

## INTRODUCTION

Fever is one of the typical early clinical symptoms of COVID-19 [1]. In most cases, patients spontaneously recover without specific treatment for COVID-19. However, some patients develop acute respiratory failure with prolonged fever requiring 2–3 weeks’ recovery with anti-viral and anti-inflammatory treatments. Herein, we report a patient with COVID-19 who had prolonged fever despite the lack of oxygen demand, who was finally diagnosed with tuberculotic lymphadenitis and HIV-1 infection.

### Case report

**Figure 1:**
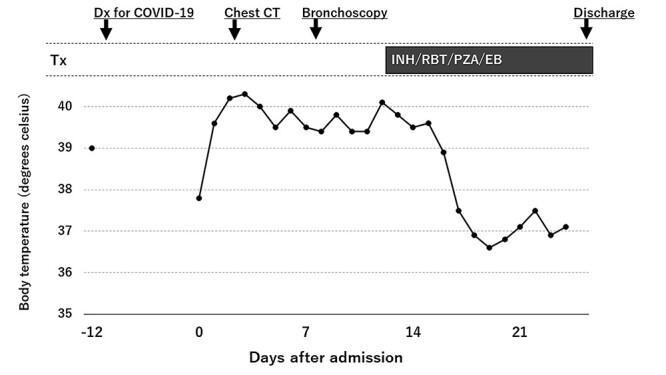
Clinical course of the patient, with daily maximum temperatures. Tx, treatment; CT, computed tomography; INH, isoniazid; RBT, rifabutin; PZA, pyrazinamide; EB, ethambutol.

**Figure 2:**
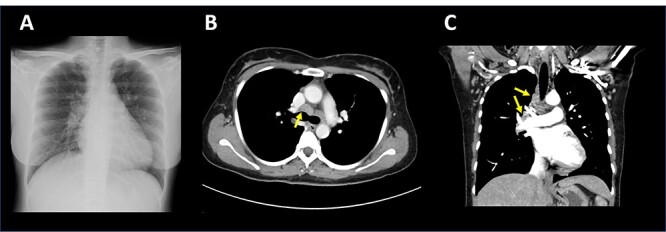
Chest X-ray on admission (**A**) and chest computed tomography on Day 7 (**B**, **C**). Enlarged lymph nodes indicated with yellow arrows.

A 33-year-old woman who visited a local public health care center because of a high fever was diagnosed with COVID-19 infection with polymerase chain reaction (PCR) using a nasopharyngeal swab (12 days before admission) (Fig. 1). She had no symptoms other than a fever of 39°C at diagnosis. However, her fever persisted for > 10 days. Therefore, she was admitted to our hospital on Day 0. She had immigrated from Nepal 1 year before the present illness. The patient lived with her husband and did not have a regular job. She denied close contact with anyone who had COVID-19 and tuberculosis; however, she sometimes serves customers at her husband’s restaurant. Her temperature, blood pressure, heart rate and peripheral oxygen saturation were 37.6°C, 104/75 mmHg, 107 bpm and 99% on room air, respectively. Physical examination revealed no abnormal findings, including lung sounds. Complete blood counts and biochemical testing showed no abnormalities, without mild anemia of 10.3 g/dL, C-reactive protein of 3.33 mg/dL, lactate dehydrogenase of 275 IU/L and D-dimer level of 6.1 μg/ml, respectively (Table 1). Nasopharyngeal PCR for SARS-CoV-2 was positive on admission. Although chest X-ray revealed no abnormal findings (Fig. 2**A**), a computed tomography scan unexpectedly showed multiple enlarged mediastinal lymph nodes (Fig. 2**B** and **C**). As deep venous thrombosis was coincidentally identified in her left femoral vein, anticoagulant treatment (rivaroxaban) was started. Her high fever continued; therefore, we suspected other chronic infections and additionally performed an HIV test and the QuantiFERON-TB Gold test (interferon gamma release assay), which were both positive (Table 2). Her CD4+ T-cell counts and HIV-1 RNA were 63 cells/μL and 1.04 × 10^6^ copies/ml, respectively. Although acid-fast bacillus testing and PCR for *Mycobacterium tuberculosis* in sputum were negative, *M. tuberculosis* was isolated from aspirated samples of mediastinal lymph node via bronchoscopy. With a diagnosis of tuberculous lymphadenitis, a four-drug antituberculosis regimen (isoniazid, rifabutin, pyrazinamide and ethambutol) was initiated on Day 13. We chose rifabutin instead of rifampicin, considering the interaction between rivaroxaban and rifampicin. The patient’s fever subsided on Day 17, and she was discharged from our hospital on Day 25. She continued therapy for 9 months. Considering the interaction with rivaroxaban, which was used for deep venous thrombosis prophylaxis, antiretroviral therapy (ART) with dolutegravir plus emtricitabine/tenofovir alafenamide were initiated on Day 35. Her CD4+ counts and HIV-RNA were 340 cells/μL and 66 copies/ml on the last follow-up date (Day 293), respectively. No immune reconstitution inflammatory syndrome (IRIS) was documented with ART.

**Table 1:** Laboratory data on admission (Day 0)

WBC	4.97	× 10E3/μL		AST	18	IU/L
Hgb	10.3	g/dL		ALT	13	IU/L
Plat	33.1	× 10E4/μL		LDH	275	IU/L
D-Dimer	6.1	μg/ml		ALP	188	IU/L
				γ-GTP	18	IU/L
				CRP	3.33	mg/dL

WBC, white blood cell; Hgb, hemoglobin; Plt, platelet; AST, asparate aminotransferase; ALT, alanine aminotransferase; LDH, lactate dehydrogenase; ALP, alkaline phosphatase; γ-GTP, gamma-glutamyl transpeptidase; CRP, C-reactive protein.

## DISCUSSION

We encountered a patient with prolonged fever who was diagnosed with mild COVID-19 infection. The patient was finally diagnosed as co-infected with HIV infection and extrapulmonary tuberculosis. Tuberculosis is a common cause of fever of unknown origin (FUO) [2]. It is reported that among all causes of FUO, 48.7% are owing to infectious diseases, among which tuberculosis is the most common, accounting for 19.5%. Differential diagnosis must always be considered in a patient with prolonged fever, even under conditions of the COVID-19 pandemic [3]. However, it is challenging for clinicians to determine when to consider the coexistence of other febrile diseases in COVID-19-positive cases. In the present case, we suspected the existence of the other diseases because the patient’s high fever had continued for > 10 days without oxygen desaturation. It is reported that the average duration of fever is 10 days in mild to moderate cases of COVID-19 infection and the duration of other symptoms is 11 days [4]. In the present case, deep venous thrombosis was identified as a complication of COVID-19 infection, which possibly contributed to the prolonged fever. However, high fever continued even after the initiation of anticoagulant treatment. Moreover, the patient’s fever rapidly subsides after the introduction of antituberculosis regimen. Taken together, prolonged fever was caused by the tuberculous lymphadenitis in this case. It is reasonable that another etiology of fever should be considered with prolonged symptoms of 11 days or longer in patients with COVID-19 who do not have pneumonia.

Interestingly, in the present case, latent extrapulmonary tuberculosis might have been reactivated owing to COVID-19 infection. A similar case has been previously reported [5]. In that case, it could be hypothesized that latent tuberculosis infection was reactivated owing to a transient CD4+ reduction with COVID-19 infection [6]. Another possibility might be that a cytokine storm with COVID-19 infection triggered the inflammatory response to latent tuberculosis infection, which is similar to unmasking IRIS after the initiation of ART in severely immunosuppressed patients with HIV. It is well known that inflammatory cytokines, such as interleukin (IL)-1, IL-6, IL-12 and tumor necrosis factor (TNF), are heavily involved during IRIS [7]. Similarly, cytokine storms caused by COVID-19 are characterized by elevation of IL-1, IL-6, IL-12 and TNF [8–10]. The QuantiFERON-TB Gold test, which frequently yields false-negative results among immunosuppressed HIV-positive individuals [11], was positive in this case and the patient’s CD4+ count was only 63 cells/μL at diagnosis. One meta-analysis showed that co-infection of tuberculosis is a significant poor prognostic factor among COVID-19-infected patients [12]. Also, it was reported that a fatal case of rapidly progressed extrapulmonary tuberculosis was triggered by COVID-19 infection in poor-adherence HIV-1-infected patient (CD4# 105/μL at COVID-19 diagnosis) [13]. Immuno-modulating effects of COVID-19 on pre-existing infectious diseases, such as tuberculosis, have been analyzed by biological models like 3D organoids [14]. Taken together, it is possible that unmasking of latent tuberculosis infection occurred owing to a cytokine storm with COVID-19 infection, although multiple replicates should be considered to conclude it.

**Table 2:** Laboratory data for microbiology

AFB blood culture (Day 2^a^)	No growth
AFB smear of sputum (Day 6^a^)	Negative
AFB culture of sputum (Day 6^a^)	Negative
PCR for M. tb of sputum (Day 21^a^)	Negative
AFB culture of aspiration from mediastinal lymph node (Day 9^a^)	*Mycobacterium tuberculosis*
QFT/IGRA	Positive^b^
HIV-screening (4th)	Positive
CD4+ T-cell counts	63/μL
HIV-RNA	1.04 × 10E6 copies/ml

^a^Date of sample collection

^b^Negative control (Nil) 0.68 IU/ml, Positive control (phytohemagglutinin) 1.29 IU/ml, TB1 1.38 IU/ml, TB2 1.99 IU/ml

AFB, acid-fast bacilli; PCR, polymerase chain reaction; QFT/IGRA, QuantiFERON-TB Gold test/interferon-gamma release assay; HIV-screening (4th), 4th generation HIV screening assay.

In conclusion, clinicians should be aware that (i) other coexisting febrile diseases are considered in mild cases of COVID-19 with prolonged fever, especially >10 days and (ii) COVID-19 infection may unmask latent opportunistic infections, such as tuberculosis, among severely immunosuppressed HIV-infected individuals.
